# ARE THE 6-MINUTE WALK TEST AND THE 2-MINUTE WALK TEST EQUIVALENT IN ACUTE AND SUBACUTE STROKE SURVIVORS IN BELGIUM AND VIETNAM: A MULTICULTURAL STUDY

**DOI:** 10.2340/jrm.v57.42665

**Published:** 2025-06-25

**Authors:** Duy Thanh NGUYEN, Chloé SAUVAGE, Claire QUESTIENNE, Michel DUCHÈNE, Duan VAN NGUYEN, Dung Tan NGUYEN, Massimo PENTA

**Affiliations:** 1 University of Medicine and Pharmacy at Ho Chi Minh City, Ho Chi Minh City, Vietnam; 2 Faculty of Human Movement Sciences, Université Libre de Bruxelles, Brussels, Belgium; 3 Service de Kinésithérapie, Hôpital Universitaire de Bruxelles, Hôpital Erasme, Brussels, Belgium; 4 Department of Rehabilitation, Da Nang C Hospital, Da Nang City, Viet Nam; 5 Department of Rehabilitation, Da Nang University of Medical Technology and Pharmacy, Da Nang City, Vietnam; 6 Institute of NeuroScience, Université catholique de Louvain, Louvain-la-Neuve, Belgium; 7 Arsalis SRL, Glabais, Belgium

**Keywords:** stroke, walk test, rehabilitation, reproducibility, cross-cultural comparison, 6MWT, 2MWT

## Abstract

**Objective:**

To test the equivalence of the 6-minute walk test and the 2-minute walk test in stroke survivors in a multicultural setting, focusing on reliability, performance, and associations with clinical and anthropometric factors.

**Design:**

Cross-sectional observational study.

**Subjects/Patients:**

238 participants (119 stroke survivors and 119 healthy individuals matched for age and sex).

**Methods:**

Participants were assessed using the 6-minute walk test and the 2-minute walk test in Belgium and Vietnam. Stroke survivors were assessed in the acute and subacute phases (17 days post-stroke) and reassessed after 1–3 days.

**Results:**

The 6-minute walk test and the 2-minute walk test showed high test–retest reliability (ICC > 0.96). The difference between the two tests was not significant in terms of walking distance reduction in stroke survivors relative to healthy participants (1.4%), not clinically meaningful for walking speed (0.026 m/s), and not significant in chronotropic response (1.6 bpm). The correlation between the 2 tests in stroke survivors was high (*r* = 0.93) and walking distance was moderately related to height, weight, and phase, and type of stroke (*p* < 0.05).

**Conclusion:**

The 6-minute walk test and the 2-minute walk test exhibited similar results in measuring the impact of stroke on walking performance. The similarity between Belgium and Vietnam further supports the clinical equivalence of both tests among acute and subacute stroke survivors.

## LAY ABSTRACT

This study compared 2 walking tests – the 6-minute walk test and the 2-minute walk test – to evaluate their usefulness for stroke survivors in a multicultural setting. A total of 238 participants were assessed, including 119 stroke survivors and 119 healthy individuals from Belgium and Vietnam. Stroke survivors were tested about 17 days after their stroke and retested after 1 to 3 days. The results showed that both tests provided similar information on walking distance, walking speed, and increase in heart rate. Both tests provided consistent results between assessments and across both countries. These results suggest that the shorter 2-minute walk test is as effective as the 6-minute walk test for measuring walking performance in stroke survivors. The consistency of results between countries underscores the potential of both tests as reliable tools to assess walking performance early after a stroke, favouring the 2-minute walk test that is more feasible and less demanding in clinical practice.

Stroke is a leading cause of physical disability, often resulting in neurological deficits like muscle weakness, spasticity, and balance impairments that significantly impact walking ability (1). Improving walking performance and cardiovascular fitness is a primary goal in stroke rehabilitation (2, 3). To accurately assess these deficits in walking capacity and evaluate the effectiveness of rehabilitation programmes, it is important to employ valid and reliable measures. In clinical practice, the 6-minute walk test (6MWT) is often used as a sub-maximal test to assess walking capacity and cardiovascular fitness (4, 5). The 6MWT has been shown to correlate with VO_2peak_ with *r* values ranging from 0.55 to 0.78 (*p* < 0.001) (6, 7) and has excellent test–retest reliability (ICC = 0.99) in measuring walking performance and cardiovascular fitness in stroke survivors (8). This test offers advantages over laboratory-based tests as it more closely resembles the ability to perform activity of daily living and does not require sophisticated equipment (9).

An alternative to the 6MWT is the 2-minute walk test (2MWT), which relies on the same procedure as the 6MWT but with a shorter execution time. The 2MWT offers the same advantages as the 6MWT and previous studies have demonstrated a strong correlation between the distances covered during both tests in healthy populations (*r* = 0.901 to 0.968) (10, 11) and in stroke survivors (*r* = 0.93) (12). Other previous studies in people poststroke reported a high correlation between the 6MWT and the 2MWT (*r* > 0.99, *p* < 0.001) (13, 14), even though the 2MWT was assessed as the first part of a longer test, which may bias the correlation with the 6MWT.

Interpreting any deficit in patients’ walking performance with the 6MWT or 2MWT in a clinical setting requires normative values. While the 6MWT is an established tool to assess walking capacity and cardiovascular fitness, normative values for this test as well as for the 2MWT exhibit cultural variations across different countries (9, 11, 15). Therefore, assessing the reliability and validity of these walking performance tests in multiple cultures requires a comparison with culture-matched normative healthy individuals.

This study aims to investigate the equivalence between the 2MWT and the 6MWT to assess walking performance in acute and subacute stroke survivors in comparison with healthy participants across 2 countries. The study investigates (1) the test–retest reliability of the 6MWT and 2MWT, (2) the participants’ performance in both tests, and (3) their relationship with clinical and anthropometric factors in stroke survivors.

## METHODS

### Participants

A cross-sectional observational study was conducted to investigate walking performance in stroke survivors in Belgium and Vietnam. Stroke survivors were included in the study if they were aged 18 years or more, and had survived a first stroke during the last 3 months with a diagnosis confirmed either by computed tomography (CT) and/or by magnetic resonance imaging (MRI). Participants had to be able to walk independently with or without a walking assistance device as measured by Functional Ambulation Category levels 4 or 5, be able to follow test instructions, and have a score ≤ 3 to the Modified Rankin Score or a score ≤ 15 to the National Institutes of Health Stroke Scale (16). Participants were assigned to 1 of 2 groups: group Walk_A_ included the individuals who could walk independently during the acute phase (i.e., within the first week) after the stroke onset; group Walk_S_ included the individuals who achieved independent walking during the subacute phase (i.e., between 8 days and 3 months) after the stroke onset. Individuals with a history of severe musculoskeletal or cardiopulmonary or other neurological disorders were not included in the study. Participants were recruited from a single hospital in each country. The study protocol was approved by the ethical committees of the Erasme Hospital (Belgium) and the Human Research Ethics Committee of C Hospital (Vietnam). All participants provided written informed consent before the assessment.

### Healthy participants matching

To compare walking performance between stroke participants and healthy controls, healthy adult participants were matched to each stroke participant from a dataset including 542 healthy individuals (11). A convenience sample of healthy adults from Belgium and Vietnam was recruited. Eligible participants were volunteers aged 18 years or more, with no history of neuromuscular, musculoskeletal, or cardiopulmonary disorders affecting walking ability. Participants had to be able to walk without any walking aid and to follow test instructions. For each stroke participant, a healthy participant from the same country and sex was identified using 4 propensity score matching (PSM) procedures: 2 countries x 2 sexes (17). Propensity scores were estimated using multiple logistic regression analysis, with the outcome being either the healthy or stroke group. The model covariates included variables that were known to influence the distance walked in the 2MWT and 6MWT, such as age, height, weight, and body mass index (BMI) (18).

### Procedures

Basic demographic characteristics and clinical information were collected from participants’ medical records. Stroke participants were instructed to rest on a chair near the starting line for at least 10 min. Participants’ heart rate was measured before and after each test with a finger pulse oximeter (MD300C63, ChoiceMed, Düsseldorf, Germany) to assess the chronotropic response to the 6MWT and 2MWT. The order of the 2MWT and 6MWT was assigned randomly. A rest period was observed between the 2 tests to allow adequate time for recovery and minimize any fatigue effect. The resting period lasted at least 15 min until the initial heart rate was reached even if it was reached before 15 min. Similar procedures were used for data collection in healthy age-matched participants.

### Six-minute and two-minute walk tests

The 6MWT and the 2MWT were administered according to the American Thoracic Society guidelines (ATS) (19). Participants were instructed to walk as far as they could. They were allowed to slow down or stop and continue to walk again after they recovered during the test period. When 1 min had elapsed, the evaluator provided standard encouragement with an even tone such as “You are doing well; you have 5, 4, 3, 2, or 1 minute(s) left”. Participants stopped at 2 min or 6 min, and the distance covered was measured to the nearest metre. The 2MWT and 6MWT were performed over a flat 30-m indoor corridor with no distracting factors according to the ATS guidelines. The corridor was marked every metre, and the turnaround points were indicated with a cone. All participants performed both walk tests without prior trial. Although it is recommended to conduct multiple trials for the 6MWT (20), participants are often tested only once in clinical practice. Therefore, the practical values from unrepeated trials in this study would be more comparable to results from daily clinical practice (21, 22).

### Reliability

To analyse test–retest reliability, stroke participants repeated the 2MWT and 6MWT after 1 to 3 days. The second assessment was conducted by the same evaluator within 1 day for acute stroke participants and within 1–3 days for subacute stroke participants to minimize the potential effects of spontaneous recovery. The order of both tests was reversed for each participant during the second session. To minimize the learning effect, participants were not informed of the test results until they had completed the test in the second session. All the evaluators had at least 5 years of experience. The same evaluator conducted the test in both sessions. The patient’s performance was recorded in an electronic file only once the test and retest were performed, hence preventing consultation with the first assessment by the examiner when performing the second.

### Data analysis

It was hypothesized that the 2MWT would demonstrate comparability to the 6MWT in acute and subacute stroke survivors in terms of: (1) test–retest reliability, (2) reduction in walking distance relative to healthy participants, (3) walking speed, and (4) chronotropic response. Furthermore, it was expected that the walking performance on the 2MWT and 6MWT would show strong correlations and that the equivalence of these tests would be consistent across participants from Belgium and Vietnam, despite cultural differences in walking patterns.

The normality of continuous variables (e.g., age, height, weight, BMI, and distances walked during the 6MWT and 2MWT) was assessed using the Kolmogorov–Smirnov test. Differences across demographic and clinical characteristics, including age, sex, height, weight, delay since stroke onset, and stroke type were assessed using a *t*-test (for comparison of means) or a χ^2^ test (for comparison of proportions) between stroke survivors and healthy participants from Belgium and Vietnam. The standardized mean differences (SMD) of the demographic variables between healthy and stroke participants was computed using Cohen’s *d* formula, defined as the difference between the means of the 2 groups divided by the pooled standard deviation.

The difference between stroke participants who took part in only 1 and both test sessions, in terms of age, sex, stroke type, side of the lesion, or distance walked in the 6MWT or the 2MWT, were assessed using *t*-tests. The test–retest reliability of walking performance in stroke participants was assessed using an intraclass correlation coefficient (ICC_2,1_) (2-way random model with measures of absolute agreement) (23). The minimum detectable change with a 95% confidence interval (MDC_95_) was determined as where *Sp* is the pooled standard deviation of test–retest trials (24). The difference in walking distance between test and retest sessions for stroke survivors was assessed using a paired *t*-test. Any difference of the walked distance at test and retest between acute and subacute stroke survivors was assessed using a 2-way ANOVA as a function of country and delay since stroke.

To compare the 6MWT and 2MWT, the mean walking speed was computed by dividing the distance walked by the duration of each test. The difference in heart rate prior to each walking test was assessed using a paired *t*-test. The chronotropic response to walking was determined by subtracting the pre-test heart rate from the post-test heart rate. The percentage of predicted maximal heart rate (%HRpred) was calculated by dividing the heart rate at the end of the walking test by the age-predicted maximal heart rate (220 minus age) and multiplying the result by 100. The differences in chronotropic response within stroke survivor group and between the 2 tests (2MWT and 6MWT) were assessed with a paired *t*-test. The equivalence of the 2MWT and 6MWT was assessed with a Bland–Altman plot (25).

Pearson’s correlation coefficient was used to explore the relationship between walking performance and linear variables such as age, height, and weight. Walking performance was compared across binomial factors such as sex, affected side, type of stroke (ischaemic vs haemorrhagic) and stroke group (Walk_A_ vs. Walk_S_) using a 2-way ANOVA as a function of country and the tested binomial factor. As walking performance differs between the 2 countries, only the effect of the tested binomial factor was considered to avoid any bias linked to the country effect. The ANOVA assumptions – including normality of residuals, homogeneity of variances (using Levene’s test), absence of outliers, and independence of observations – were assessed. The level of significance for all statistical analyses was set at 0.05. All analyses were performed using the R language for statistical computing (version 4.0.5; R Foundation for Statistical Computing, Vienna, Austria).

## RESULTS

A total of 119 consecutive individuals poststroke were enrolled including 33 subjects (27.7%) in Belgium and 86 subjects (72.3%) in Vietnam. At the time of assessment, the median delay since stroke was 17 days (IQR = 5 to 37.5 days) and only 3 participants were using an assistive device for walking (1 patient used a crutch in Belgium and 2 patients used a 3-point cane in Vietnam). Following the inclusion criteria, all stroke participants were classified at FAC level 4 or 5. All participants had a Modified Rankin Score (mRS) of 2 or 3, consistent with the inclusion criteria (mRS ≤ 3). All participants were classified as having a mild to moderate stroke. All continuous variables were normally distributed except for the time delay since stroke onset. No statistically significant differences were observed between stroke participants from Belgium (*n* = 33) and Vietnam (*n* = 86) across key demographic (age and sex) and clinical characteristics; only height (mean of 1.72 m in Belgium, and of 1.61 m in Vietnam, *p* < 0.001) and weight (mean of 76.6 kg in Belgium, and of 61.6 kg in Vietnam, *p* < 0.001) differed between countries. A matched sample of 119 healthy participants was obtained from the PSM with no significant demographic differences. The SMDs of the demographic variables between healthy and stroke participants are presented in Table I. The overall mean SMD across all variables was 0.037, indicating that the differences between the stroke survivors and the matched healthy participants are negligible in terms of demographic and anthropometric characteristics (26).

**Table I:** Characteristics of stroke participants and matched healthy participants

Characteristic	Stroke participants (*n* = 119) Mean (SD)	Matched healthy participants (*n* = 119) Mean (SD)	Standardized mean difference
Country, *n* (%)			
Belgium	33 (27.8)	33 (27.8)	
Vietnam	86 (72.2)	86 (72.2)	
Age (years)	60.8 (13.5)	60.1 (14.0)	0.047
Sex, *n* (%)			
Female	38 (31.9)	38 (31.9)	
Male	81 (68.1)	81 (68.1)	
Height (m)	1.64 (0.1)	1.64 (0.1)	0.022
Weight (kg)	65.7 (12.9)	65.3 (13.3)	0.033
Body mass index (kg/m^2^)	24.4 (3.4)	24.2 (3.3)	0.049
Type of stroke, *n* (%)			
Ischaemic	102 (85.7)		
Haemorrhagic	17 (14.3)		
Affected side, *n* (%)			
Right	52 (43.7)		
Left	67 (56.3)		
Stroke group, *n* (%)			
Walk_A_	41 (34.5)		
Walk_S_	78 (65.5)		

SD: standard deviation; WalkA: independent walk in acute phase (< 8 days poststroke); WalkS: independent walk in subacute phase (8 days to 3 months poststroke).

Among the 119 poststroke survivors who performed both walking tests for the first time, 67 participants were reassessed a second time to evaluate the test–retest reliability. There were no significant differences between stroke participants who were assessed only once and those who were reassessed in terms of age, sex, stroke type, and side of the lesion, except for the delay from stroke onset to the first test (19.7 days for participants who took part in only 1 test, 30.4 days for participants who attended both tests, *p* = 0.021). Notably, the distance walked in the 6MWT or the 2MWT was not significantly different between stroke participants who were assessed once and those who were reassessed (*p* > 0.12). Reasons for dropout includ-ed early discharge, transfer to another hospital, or a preference not to participate in the second assessment session.

**Fig. 1:**
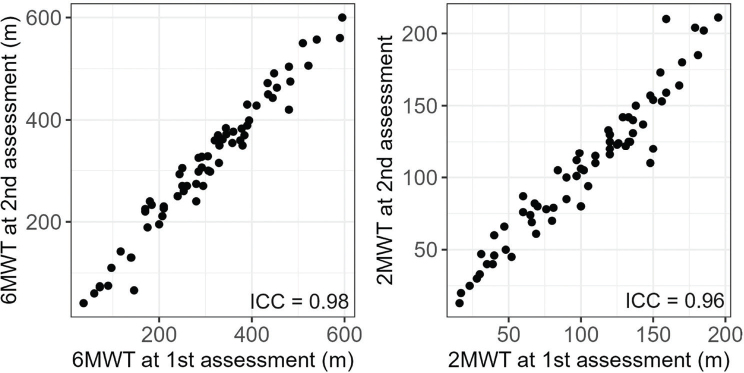
Distance walked during the first and second assessment with the 6-Minute walk test (6MWT) and with the 2-minute walk test (2MWT). The intraclass correlation coefficient (ICC) shows excellent test–retest reliability for both tests in stroke participants (*n* = 67).

The test–retest reliability for walking distance was high for the 6MWT (ICC = 0.98, 95% CI = 0.96–0.99) and for the 2MWT (ICC = 0.96, 95% CI = 0.93–0.98) (see Fig. 1). The standard error of measurement (SEM) was 19.9 m for the 6MWT and 7.2 m for the 2MWT, leading to an MDC_95_ for walking distance of 55.9 m for the 6MWT and of 20.2 m for the 2MWT. Despite the high test–retest reliability of the 6MWT and 2MWT, a statistically significant increase was observed in walked distance between the 2 assessments for the 6MWT (*p* = 0.002) and for the 2MWT (*p* = 0.015), with mean (SD) distances walked of 280 (124.0) m for the first and 313 (134.0) m for the second 6MWT and of 97 (43.8) m and 107 (47.9) m for the 2MWT, respectively. There was no significant difference in the mean change in walking distance between test and retest sessions for participants in the acute and subacute phases (*p* > 0.27).

**Fig. 2:**
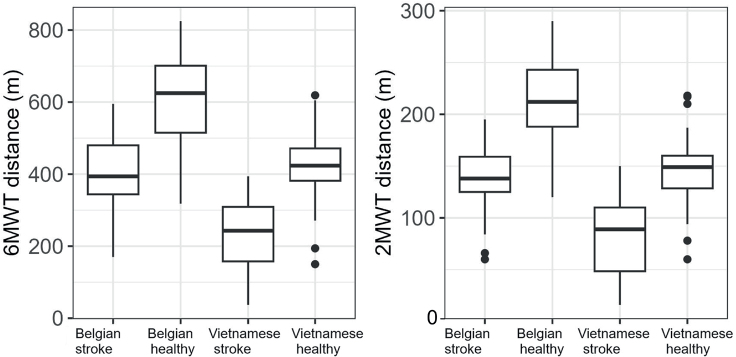
Walked distances in stroke participants (*n* = 119) and healthy matched participants (*n* = 119) in Belgium and Vietnam during the 6-Minute Walk Test (6MWT) and the 2-Minute Walk Test (2MWT).

The walking performance of stroke and matched healthy participants is reported in Table II. Healthy matched participants walked further than stroke participants during the 6MWT and 2MWT regardless of their country (all *p*-values < 0.001). The walking distance is higher in Belgium than in Vietnam for both stroke and healthy participants, as shown in Fig. 2. Notably, whichever country, the reduction in walking performance of stroke participants compared with healthy participants is not significantly different for the 6MWT and the 2MWT (1.4%; 95% CI –0.8 to 3.5; *p* = 0.209).

**Table II:** Walking performance in stroke participants and matched healthy participants

Distance	Stroke participants (*n* = 119) Mean (SD)	Matched healthy participants (*n* = 119) Mean (SD)
6MWT (m)		
Both countries	280 (123.5)	470 (119.5)
Belgium	402 (105.0)	594 (122.0)
Vietnam	233 (95.1)	423 (77.2)
2MWT (m)		
Both countries	97 (43.8)	164 (42.9)
Belgium	138 (33.4)	210 (44.1)
Vietnam	81 (36.2)	147 (26.2)

SD: standard deviation; 6MWT: 6-Minute Walk Test; 2MWT: 2-Minute Walk Test.

**Fig. 3:**
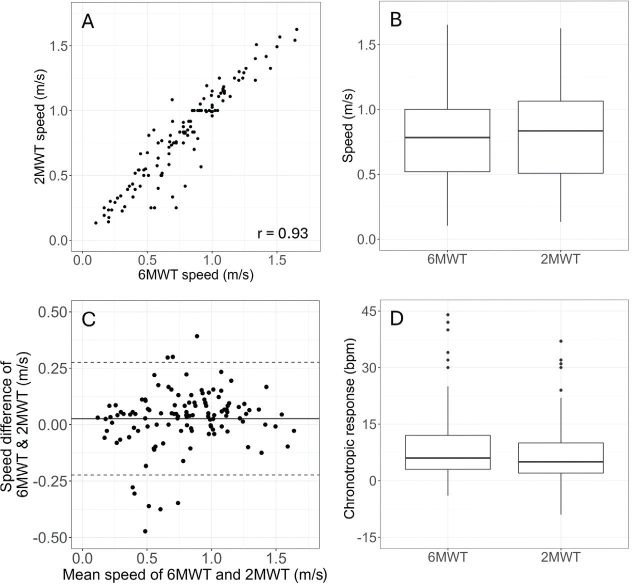
Illustration of the equivalence of the 6MWT and 2MWT to assess walking performance in mild to moderate stroke participants (*n* = 119), according to (A) the correlation between the walking speed during the 6MWT and the 2MWT; (B) the box plot of the median walking speed during the 6MWT and the 2MWT; (C) the agreement between the walking speed measured during the 6MWT and the 2MWT relative to the mean difference (plain line) and agreement lines set at ±1.96 SD from the mean (dashed lines); and (D) the box plot of the chronotropic response during the 6MWT and 2MWT.

Stroke participants walked at a mean speed of 0.805 m/s in the 2MWT, which was slightly faster than the 6MWT (0.778 m/s), although this difference (0.026 m/s; 95% CI 0.003 to 0.049; *p* = 0.026) remained within the measurement error as indicated by the MDC_95_ of 0.031 m/s for the 6MWT and of 0.061 m/s for the 2MWT. The correlation between participants’ walking speed in both tests remained high and statistically significant (*r* = 0.93, *p* < 0.001), also supporting the equivalence in walking speed of both tests shown by the Bland–Altman plots in Fig. 3.

According to the test procedures, the heart rate of the stroke participants was not significantly different before the 6MWT and before the 2MWT (0.7 bpm; 95% CI –0.2 to 1.6; *p* = 0.135). All the stroke participants reached their initial resting heart rate during the rest period that preceded each test (*p* = 0.61 for the 6MWT and *p* = 0.26 for the 2MWT). The mean heart rate after the tests in both countries reached a value of 95 (19.2) bpm for the 6MWT and of 93 (17.6) bpm for the 2MWT. These heart rates after the walking tests reflected a %HRpred of 56 (9.7) % and 56 (9.5) % for the 6MWT and 2MWT, respectively. Whichever the country, there was no significant difference in the chronotropic response after the 6MWT and the 2MWT (1.6 bpm; 95% CI –0.1 to 3.0; *p* = 0.107), as also illustrated in Fig. 3.

The correlation between the distance walked by stroke participants in the 2 tests was high and statistically significant (*r* = 0.93, *p* < 0.001). The relations between the distance walked and clinical and anthropometric characteristics are presented in Table III. There were weak to moderate significant correlations between walking distance and height and weight. Notably, age and sex demonstrated no significant correlation with walked distance in stroke participants. However, there was a disparity in the distance walked between the 2 types of stroke. The ischaemic group walked 289.3 m in the 6MWT and 99.8 m in the 2MWT, which is significantly higher than the 225.8 m and 77.3 m, respectively, achieved by the haemorrhagic group (all *p*-values < 0.05). Participants with minor strokes were able to walk earlier and were classified as FAC level 4 shortly after the stroke, whereas those with moderate strokes participated in the study later, during their subacute phase. The participants in group Walk_A_ walked a significantly longer distance (327 m for the 6MWT and 113 m for the 2MWT) than the participants in group Walk_S_ (255 m for the 6MWT and 88 m for the 2MWT, *p* < 0.001 for both tests). The residuals for all ANOVA models were normally distributed, and Levene’s test confirmed homogeneity of variances (all *p* > 0.05). No outliers were detected, and the observations were independent.

**Table III:** Relationship of distance of 6-minute walk test (6MWT) and 2-minute walk test (2MWT) with personal factors in stroke participants (*n* = 119)

	6MWT	*p-*value	2MWT	*p-*value
Age	*r* = –0.03	0.725	*r* = –0.05	0.591
Sex	*r* = –0.04	0.681	*r* = –0.08	0.403
Height	*r* = 0.49	< 0.001	*r* = 0.50	<0.001
Weight	*r* = 0.35	< 0.001	*r* = 0.32	<0.001
Type of stroke (ischaemic vs haemorrhagic)	*F* = 6.196	0.014	*F* = 5.921	0.017
Affected side (right vs left)	*F* = 0.214	0.644	*F* = 0.009	0.924
Stroke group (Walk_A_ vs Walk_S_)	*F* = 17.540	< 0.001	*F* = 17.087	<0.001

Reported statistics are as follows: *r* for Pearson correlations, *F*-value for two-way ANOVA. The strength of any relationship was assessed as: absent or weak relationship for *r* < 0.25; weak to moderate for 0.25 ≤ *r* < 0.5; moderate for 0.5 ≤ *r* < 0.75; or strong relationship for *r* ≥ 0.75.

## DISCUSSION

This study aimed to characterize the assessment of walking performance after a mild to moderate stroke in Belgium and Vietnam and revealed several key insights. Both the 6MWT and 2MWT exhibited high test–retest reliability in stroke survivors. The 6MWT and 2MWT were comparable in measuring the impact of stroke on walking distance, speed, and chronotropic response relative to matched healthy participants. Strong correlations between distances covered in the 6MWT and 2MWT were observed in acute and subacute stroke survivors.

In terms of reliability, both the 6MWT and 2MWT demonstrated high test–retest reliability in acute and subacute stroke survivors who could walk independently, with ICC values of 0.98 and 0.96, respectively. The calculations of SEM and MDC_95_ indicated minimal measurement errors, suggesting that both tests consistently yield reproducible measurements in both countries, in stroke survivors who can walk independently. These findings align with previous studies, which reported excellent reliability of the 6MWT in individuals poststroke in subacute (ICC = 0.97) (27) and in chronic stages (ICC = 0.98 to 0.99) (8, 27). The test–retest analysis used a complete-case approach, including just over 50% of participants (67 among 119 stroke survivors). Those who completed only 1 session had a shorter stroke onset delay (19.7 vs 30.4 days, *p* = 0.021). This difference likely occurred because participants who regained walking ability earlier during the acute stage were discharged sooner and, consequently, did not participate in the second test session. As a result, participants who recovered more quickly or had milder impairments may be underrepresented in the test–retest analysis.

The MDC_95_ in this study was 55.9 m for the 6MWT and 20.2 m for the 2MWT. These values were comparable to those reported in previous studies, which reported an MDC_95_ of 54.1 m for the 6MWT in subacute stroke survivors (27) but were higher than the 13 m reported for the 2MWT in chronic stroke survivors (28). Variability in stroke stage, level of walking capacity, and difference in sample size could explain these discrepancies compared with previous studies (29). The MDC in this study suggests the potential use of these tests to measure the effects of treatment with acceptable measurement errors.

This study used a multicultural setting to compare the walking performance of stroke participants to the 6MWT and the 2MWT with healthy participants matched according to sex, age, and anthropometric characteristics. The results showed that stroke participants walked significantly slower than healthy matched participants during the 6MWT, covering 61% of the distance covered by the healthy participants. This observation confirms a previous report where mild chronic stroke individuals walked 63% of the predicted distance (30). This finding also emphasizes the importance of using country-specific normative values to accurately interpret individuals’ performance (9, 15, 31). Noticeably, despite the observed difference in walking distance between countries, the discrepancies in walking distance between stroke and healthy participants remained similar for both the 6MWT and the 2MWT (*p* > 0.05). This similarity suggests a consistent effect of stroke-related impairments on walking performance that can be captured equally by the 6MWT and the 2MWT in acute and subacute stroke survivors.

There was a strong correlation between the distances between the 6MWT and the 2MWT in stroke survivors. This finding is similar to previous research on both acute (*r* = 0.99) (13) and chronic stroke individuals (*r* = 0.93) (31). Previous studies have examined the correlation between the distance covered in the first 2 min of the walking test and the entire 6 min test (13, 14), whereas the current study reported the correlation of the 2 separate tests. In the same way, our results showed that the correlation between walking speeds in both tests remained high and statistically significant (*r* = 0.93, *p* < 0.001), suggesting equivalence of the speed of 2 tests. Although a significant difference in speed was observed between the 6MWT and 2MWT (mean difference: 0.026 m/s), its impact on performance was minimal. This difference translates to 3 m in the 2MWT and 9 m in the 6MWT, which is not clinically meaningful as it represents less than 16% of the MDC_95_. These findings support the equivalence of both tests in our sample of stroke participants. The Bland–Altman plot did not reveal any systematic difference in the speed during both tests, suggesting that the 2MWT is equivalent to the 6MWT and can effectively replace the 6MWT to analyse walking capacity and evaluate the effectiveness of rehabilitation programmes in acute and subacute stroke participants who can walk independently.

The heart rate at the end of the 6MWT in the current study was 56% of the age-predicted maximal heart rate, corresponding to the 58% reported in a previous study (14), suggesting that participants exhibited a similar chronotropic response during the 6MWT in both studies. Our results showed that the heart rates at the end of the 2MWT and the 6MWT did not differ significantly (*p* = 0.145), and the mean difference in chronotropic response between the tests was not significant at 1.6 bpm (*p* = 0.107). These findings confirm a previous study concerning the change in heart rate during a 6MWT in chronic stroke participants, indicating that a substantial increase (of 36%) during the first 2 min was followed by a smaller increase (7%) during the subsequent 4 min (8). The equivalence in chronotropic response between walking tests suggests that the additional 4 min of the 6MWT do not provide further information regarding walking performance compared with the 2MWT in acute or subacute stroke survivors. Nevertheless, shorter performance tests may still overestimate walking endurance (13). Therefore, depending on the specific treatment objective and the patient’s clinical profile, it is important to consider this potential overestimation when choosing the most suitable walking performance assessment.

Age, sex, and anthropometric factors are significantly correlated with walking distance in healthy individuals (11, 18, 33). In the current study, no significant correlations were observed with age or sex for stroke participants and weak to moderate correlations were found with height and weight. These findings are consistent with a previous study on stroke individuals (14), which reported that demographic factors, such as age and sex, had minimal influence on the distance walked in stroke participants. Clinical factors related to stroke, such as the presence of hemiparesis, balance disorders, or spasticity, are likely to have a greater influence on walking performance than demographic factors in stroke survivors (34). The affected side did not relate to walking performance with either test, unlike the type of stroke and the time required to acquire independent walking ability. These results are in line with a previous study, showing that the side of the lesion did not affect the distance walked in either the 6MWT or the 2MWT (14). Participants with haemorrhagic stroke showed significantly lower walking performance compared with those with ischaemic stroke. This confirms other findings where a haemorrhagic stroke was associated with a greater initial severity (35, 36) and poorer long-term outcomes than an ischaemic stroke (37). Additionally, stroke survivors assessed in the acute phase demonstrate higher walking performance than those assessed in the subacute phase. This is most likely due to the difference in severity of the stroke among participants. Indeed, participants with a minor stroke were classified as FAC level 4 and recruited in the study earlier after their stroke, whereas those with a moderate stroke were recruited later during their subacute phase. These results suggest that clinicians should consider the type and severity of stroke and the individual’s impairments when establishing and assessing rehabilitation programmes to improve walking performance (34, 38, 39).

To our knowledge, there is no study that has compared the walking performance between stroke participants with matched healthy participants in both the 6MWT and 2MWT in multicultural settings. The fact that stroke survivors were from different countries (Belgium and Vietnam), where populations usually walk at different speeds (11), further supports the equivalence of the 6MWT and the 2MWT in our study.

### Strengths and limitations

The strength of this study is that it utilized an adequate sample size and adhered to the ATS recommendations for conducting walking tests. The generalizability of the results may be limited due to the characteristics of the stroke participants and the fact that data were collected from a single centre in each country. All participants were independent walkers, with only 3 using walking aids, which suggests a high-functioning sample that does not fully represent the broader stroke population in both countries. Another limitation is that unrecorded participants’ medication, such as beta-blockers or calcium channel blockers, may have affected heart rate measurements, potentially influencing our findings.

### Conclusion

This study provides evidence supporting the use of the 2MWT as an easier and equivalent alternative to the 6MWT in assessing walking performance in acute and subacute stroke survivors. The study found that the 2MWT had a high test–retest reliability, equivalent to 6MWT in measuring stroke-related impairments, and highlighted its clinical utility in stroke assessment. Its shorter duration and comparable reliability make it a valuable tool for clinicians and researchers, particularly in settings where time and resources are limited.

## Clinical trial registration number

Belgian site: B4062021000237; Vietnamese site: 76/CT-HDDD.
